# Design of a Fixed IF Down-Conversion Double-Balanced Mixer for UHF Band Applications

**DOI:** 10.3390/s25030608

**Published:** 2025-01-21

**Authors:** Trusha Kared, Helga Silaghi, Matthias Rudolph, Andrei Silaghi, Ulrich L. Rohde

**Affiliations:** 1 Radio Frequency and Microwaves Techniques Department, Brandenburg University of Technology Cottbus, 03046 Cottbus, Germany; tskared103@gmail.com (T.K.); mattias.rudolph@gmail.com (M.R.); dr.ulrich.l.rohde@gmail.com (U.L.R.); 2 Synergy Microwave Corporation, 201 McLean Blvd, Paterson, NJ 07504, USA; 3 Department of Control Systems Engineering and Management, University of Oradea, 410087 Oradea, Romania; hsilaghi@uoradea.ro; 4 Measurements and Optical Electronics Department, Politehnica University Timisoara, 300223 Timișoara, Romania; 5 Institute for Informatics, Universität der Bundeswehr München, 85579 Neubiberg, Germany

**Keywords:** military radar, satellite communication (SATCOM), cellular base stations, mixer, lo balun, impedance matching

## Abstract

This paper presents a new SiGe HBT-based high dynamic range double-balanced down-conversion differential mixer. Operating within the 0.5 GHz to 1.8 GHz range, the suggested mixer is appropriate for a variety of applications, such as cellular base stations, satellite communication (SATCOM), and military radar. The down-conversion mixer is made up of a single-ended to a differential-balanced radiofrequency (RF) stage, a dual feedback linearization for the RF stage, a local oscillator (LO) balun, LO mixing cores, and a fixed intermediate frequency (IF)-tuned circuit connected between two outputs to serve as a load at 145 MHz. Compared to earlier research in the literature, the measured SSB noise figure is approximately 7 dB ± 0.4 dB, and the measured conversion gain is approximately 12 dB ± 1 dB for a full band of operation. The mixer achieves a good return loss of over 8 dB for an RF and LO port in the desired band and a measured return loss of over 18 dB at 145 MHz and IF frequency. Furthermore, the design achieved an RF-to-IF isolation of greater than 35 dB, LO feedthrough, and an LO leakage isolation of better than 50 dB. Lastly, the measured third-order intercept point was found to be +4.7 dBm, and the 1 dB compression point was approximately −8 dBm. These results demonstrate good linearity performance.

## 1. Introduction

A mixer combines a radiofrequency (RF) input signal at frequency $f_{RF}$ and a local oscillator (LO) signal at frequency $f_{LO}$ to produce an intermediate frequency (IF) output signal containing the sum and difference frequencies, $f_{RF}$ ± $f_{LO}$. Either the sum ($f_{RF}$ + $f_{LO}$) or the difference ($f_{RF}$ − $f_{LO}$) frequency is selected for further processing using a bandpass filter [1,2,3].

Mixers are frequently classified as passive or active. Passive mixers can be employed to generate an IF output for an LO driving signal by using passive switches. In contrast to active mixers, passive mixers do not provide a positive conversion gain (CG) [2,3]. However, passive mixers are straightforward. They require a high LO drive level but are highly linear and have a good noise figure (NF), which is typically the same as conversion loss and consumes zero power. Unlike passive mixers, active mixers offer exceptional CGs, effective port isolation, minimal NF, and require less LO drive. Therefore, active mixers are favored over passive mixers when all of these factors are taken into account.

In the past years, literature has presented several types of mixers [4,5,6,7,8,9,10,11].

For instance, Ref. [5] proposes a broadband double-balanced mixer that operates at 1–6 GHz. Two techniques are proposed to enhance the mixer’s performance. First, at high frequencies, loading capacitors improves the amplitude and phase imbalance of the balun. Second, by cascading a first-order LC filter at the intermediate-frequency port, RF and LO signal leakage at the IF port is decreased by around 8 dB and 7 dB, respectively. With a 1.13 mm × 0.95 mm chip size and 34 dB of isolation between the IF and RF ports, the device produces 13.5 dBm of input power at the 1 dB compression point. In order to build the chip, the GaAs pHEMT technique is employed [5].

Based on reformed particle swarm optimization, the authors of [8] suggest an up-conversion mixer circuit for radar. The suggested approach assists in resolving the issues of premature convergence and local optima in conventional particle swarm optimization. Additionally, the paper [8] presents new circuit designs based on the two-fold transconductance route, a linearity-enhancing approach. The suggested optimal mixer obtains a measured conversion gain of 2.5 dB. The mixer proposed in the aforementioned study has a good linearity and a 1 dB compression point of 4.2 dBm. The noise figure of the suggested mixer is roughly 3.1 dB [8].

In paper [9], a novel linearization method for active mixers is proposed, which takes advantage of the “reverse uplift” phenomena to significantly increase the input and output 1 dB compression points. This technique is used to design and build a 130 nm SiGe BiCMOS ultra-high-linearity double-balanced down-conversion mixer with a broad IF bandwidth. A Gilbert cell, two phase-adjusting inductors, and an output network based on a Marchand balun are all components of the suggested mixer. According to the measurement results, the mixer has the best linearity performance in mixers under silicon-based processes, whether active or passive, with an IP1dB of +7.2~+10.1 dBm and an average OP1dB of +5.4 dBm. In addition, a wide 8 GHz IF bandwidth between 3 and 11 GHz was achieved [9].

Finally, a reconfigurable, image-rejected down-conversion mixer, based on the SiGe 8 HP technology, has been presented in [10]. The suggested mixer is appropriate for software-defined radio applications and operates within the 0.9–13.5 GHz range. The conversion mixer is made up of a tunable filter section, a resistive biased radiofrequency section, and double-balanced Gilbert-cell mixer core sections. The mixer achieves a good return loss |*S*11| of <−10 dB for the whole operating band. Additionally, at maximum frequency, the design achieves an outstanding CG of 22 dB, an NF of 2.5 dB, and an image-rejection ratio of 30.2 dB. Finally, moderate linearity performance is shown with a third-order intercept point of −3.28 dBm and a 1 dB compression point of −13 dBm, respectively [10].

This study presents a novel SiGe HBT-based differential double-balanced down-conversion mixer. The proposed mixer is specifically designed to address key challenges studied in [4,5,6,7,8,9,10,11] in terms of conversion gain, low noise figure, excellent linearity, wide dynamic range, and port-to-port isolation. Compared to existing designs, this mixer achieves impressive performance metrics, such as a single-sideband (SSB) noise figure of approximately 7 dB ± 0.4 dB, a conversion gain of 12 dB ± 1 dB, and an RF-to-IF isolation exceeding 35 dB, and LO-to-RF and LO-to-IF isolation are better than 50 dB in the frequency band of 0.5 GHz–1.8 GHz. Furthermore, it demonstrates excellent linearity, with a third-order intercept point (IP3) of +4.7 dBm and a 1 dB compression point of −8 dBm.

The design combines several state-of-the-art features, including a single-ended-to-differential signal conversion by eliminating the need for a transformer at the RF stage, and a dual feedback linearization technique to improve nonlinear transfer function to achieve better impedance matching while minimizing signal distortion, resulting in a highly efficient and more linear mixer architecture. To avoid cross-talk interference of LO and RF signal, symmetrical active device layout and multilayer board technology are used, which significantly reduced LO to RF feedthrough, resulting in improved port-to-port isolation.

The present paper is structured in the following way. Section 1 discusses general topics related to mixers and introduces a double-balanced mixer operating principle in Section 2. Section 3 focuses on the design and optimization of a fully monolithic double-balanced down-conversion Gilbert-cell mixer. Section 4 presents simulation results (linearity, noise, and gain calculations) and looks at the experimental validation of the proposed mixer. Concluding remarks are given in the final part of the paper.

## 2. The Design Methodology

**Figure 1:**
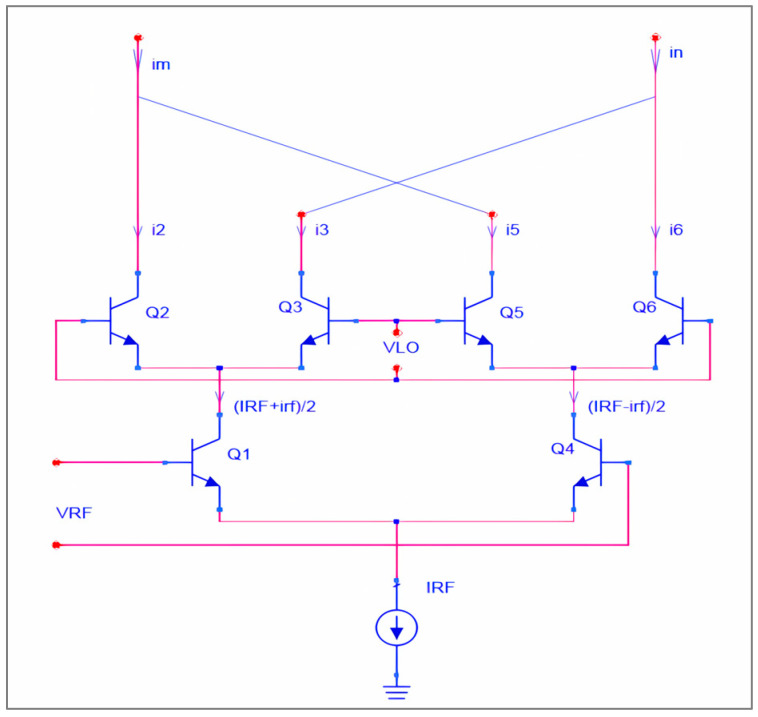
Double-balanced Gilbert-cell mixer.

A double-balanced mixer optimally combines RF and LO signals while inhibiting significant LO components appearing at the IF output. The method utilizes two cross-coupled single-balanced networks to eliminate in-phase and out-of-phase local oscillator components prior to the current-to-voltage conversion stage. Figure 1 illustrates the configuration of the Gilbert cell used for this mixer; the differential output current $i_{out}$ can be written as follows:

(1) $$i_{out}=i_{m}-i_{n}=I_{RF}\tanh\left(\frac{v_{RF}}{2V_{T}}\right)\tanh\left(\frac{v_{LO}}{2V_{T}}\right)$$

Taking into account both large and small signal components in the $i_{out}$ of the RF input pair and calculating the output currents, we obtain the following:

(2) $$i_{m}=\left(\frac{I_{RF}}{2}\right)+(i_{rf})\tanh\left(\frac{v_{LO}}{2V_{T}}\right)$$

(3) $$i_{n}=\left(\frac{I_{RF}}{2}\right)-(i_{rf})\tanh\left(\frac{v_{LO}}{2V_{T}}\right)$$

From Formulas (2) and (3), the differential output current can be written as

(4) $$i_{out}=\frac{i_{m}-i_{n}}{2}=i_{rf}\tanh\left(\frac{v_{LO}}{2V_{T}}\right)$$

The double-balanced mixer output current expression does not include the large-signal component, $I_{RF}$, according to Formula (4). Within differential pair matching limits, the double-balanced topology effectively eliminates the DC component of bias current at the IF port; however, the presence of extra devices at the RF port may result in a higher noise figure.

## 3. Proposed Down-Conversion Mixer Design

**Figure 2:**
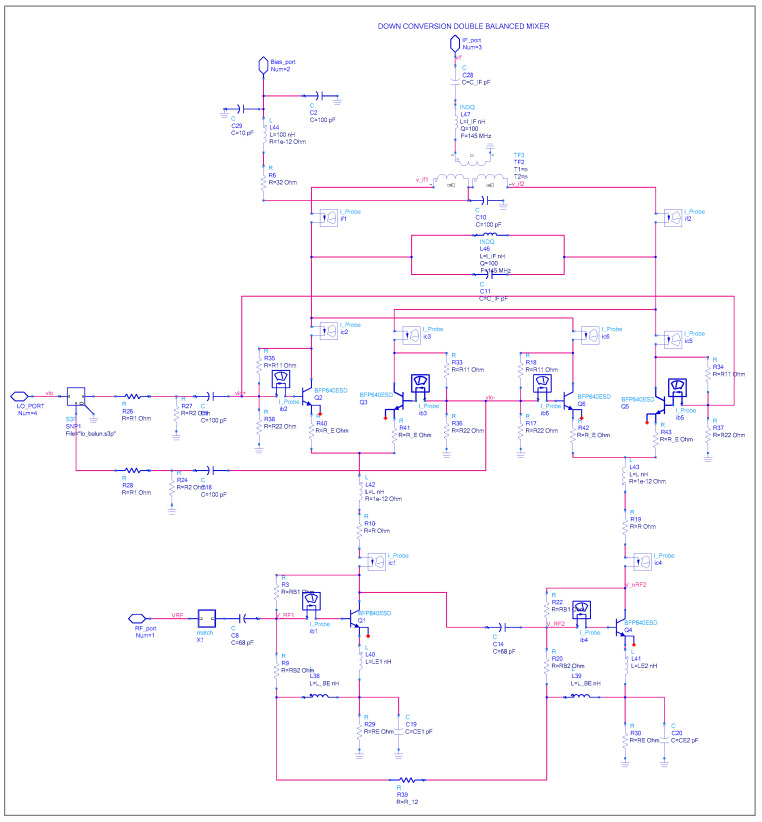
Active differential double-balanced down-conversion mixer.

The proposed differential down-conversion double-balanced architecture is shown in Figure 2. It comprises four Infineon BFP640 devices functioning as switches for the LO feed and two BFP840 devices designated for differential RF input. The Infineon BFP840 device was selected for the RF stage due to its higher $f_{T}$ frequency and lower internal base resistance. We need the higher LO drive for the local oscillator stage. The output 1 dB compression point for the BFP640 device is roughly +11 dBm, whereas for BFP840, it is about 5 dBm. The design is based on coupled differential amplifiers, enabling the Gilbert cell to achieve high gain, low noise, wide bandwidth, and low power consumption, making it suitable for monolithic IC implementation.

This work presents the design and the optimization of a completely monolithic double-balanced down-conversion Gilbert-cell mixer with a single-ended RF input and an IF-tuned circuit with differential output at 145 MHz. The local oscillator frequency sweep ranges from 355 MHz to 1855 MHz and is applied differentially through a balun to the four local oscillator transistors. The mixer is designed for a frequency range of 500–2000 MHz, with the RF signal being fed differentially (single-ended to differential conversion) to the RF transistor, as seen in Figure 2. Each RF stage draws about 10 mA, and the supply voltage is 4 V. So, the total power consumption of the circuit is approximately 80 mW (Total power consumption = 2 × 10 mA (each branch draws 10 mA) × 4 V (supply voltage)).

Each RF stage incorporates multiple feedback mechanisms to address the nonlinearity present in transistors. Simultaneously, the series feedback from the second stage to the first stage serves to correct the phase shift between the two RF currents and enhances the dynamic range of the mixer. The circuit employs dual feedback linearization at each RF stage, combining series inductive feedback (at the input side) and shunt resistive feedback (at the output side). Series feedback stabilizes input current, while shunt feedback controls output voltage, hence improving the nonlinear transfer characteristics of the transistor, reducing distortion, and ensuring a more linear performance of the circuit.

The mixer circuit is less influenced by the source due to the high input impedance of the series feedback. It improves impedance matching at the input, reducing noise contribution at the RF stage, hence providing a high signal-to-noise ratio at the input. Shunt feedback limits noise propagation at the output as it decreases the output impedance, making it easier for the circuit to drive a load. This improves signal transfer to the next stage with minimal loss. The combined effect results in enhanced linearity, a wide dynamic range, and a low noise figure. A 1000 MHz RF frequency and a fixed IF frequency of 145 MHz are used in the design to achieve the best possible performance in terms of conversion gain, noise figure, linearity, and dynamic range.

Two voltage-dependent current sources, each with the same bias current, are used in the double-balanced mixer. The IF-port current alternates between ${g_{m}V}_{RF}$ and −${g_{m}V}_{RF}$, and the DC bias current $I_{bias}$ is always balanced as it passes through both loads. Thus, the differential output voltage $V_{IF}$ is independent of the biasing current. The IF-port current can be calculated as follows:

(5) $$I_{IF}={\;{\;g}_{m}V}_{RF}\left\{\frac{2}{\pi }{cos(\omega }_{RF}-\omega_{Lo})+\frac{2}{\pi }{cos(\omega }_{RF}+\omega_{Lo})+\frac{2}{3\pi }{cos(\omega }_{RF}-{3\omega }_{Lo})+\frac{2}{3\pi }{cos(\omega }_{RF}+{3\omega }_{Lo})+\cdots \right\}$$

Therefore, the conversion gain of the Gilbert-cell mixer is as follows:

(6) $$GC=\frac{2}{\pi }g_{m}R_{L}$$

Expression (6) shows that the gain of the double-balanced mixer is the same as that of a single-balanced mixer.

Under ideal switching conditions, the optimal conversion gain can be achieved. The switching pairs are not ideal in practice. During the switching time period $t_{switch}$, both switches are on, transforming the switching pairs into differential amplifiers. When |$V_{LO}$| is less than $4V_{t}$, the conversion gain that considers this non-ideality [12] is expressed as follows:

(7) $$GC≅\frac{2}{\pi }\frac{sin(\pi f_{LO}t_{switch})}{\pi f_{LO}t_{switch}}g_{m}R_{L}$$

According to Formula (7), switching time can be reduced by increasing the LO voltage or lowering the switching voltage in order to increase conversion gain. Higher LO drive may lead to saturation in transconductance stages and cause the RF signal to swing beyond the pre-described linear range, which results in lower transconductance $g_{m}$ and consequently, lowers linearity and gain. Switching does not require high voltage. Hence, it reduces switching time while keeping a lower power level for LO, which is highly beneficial in low power applications [12,13,14,15].

### 3.1. Single-Ended to Balanced RF Stage Driver Design and Optimization

**Figure 3:**
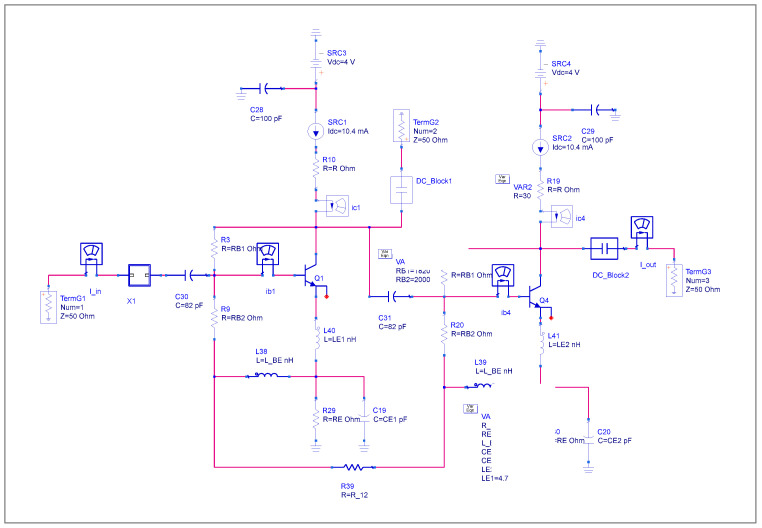
RF stage amplifier.

Implementing a Gilbert-cell mixer presents a challenge in using differential amplifiers within the cell itself. The optimal differential driver of the RF port is the most critical trade-off. Here, a better solution is employed in place of costly and potentially asymmetrical transformers, such as those found in diode mixers. Such a transformer is costly and difficult to implement at the RF stage. The proposed solution is to use first a pre-driver stage to convert a single-ended line to a balanced line [3]. As shown in Figure 2, we use the phase shift of 180° of the first RF stage transistor and drive the second transistor. Ideally, the first RF common emitter stage will generate a 180° phase shift between the base and the collector. At the second stage, the base-collector phase shift will be converted back to 0°. In reality, the phase shift between two transistors is never ideal. Figure 3 shows the simulated stand-alone RF stage to analyze the single-ended RF signal behavior to balanced signal conversion.

**Figure 4:**
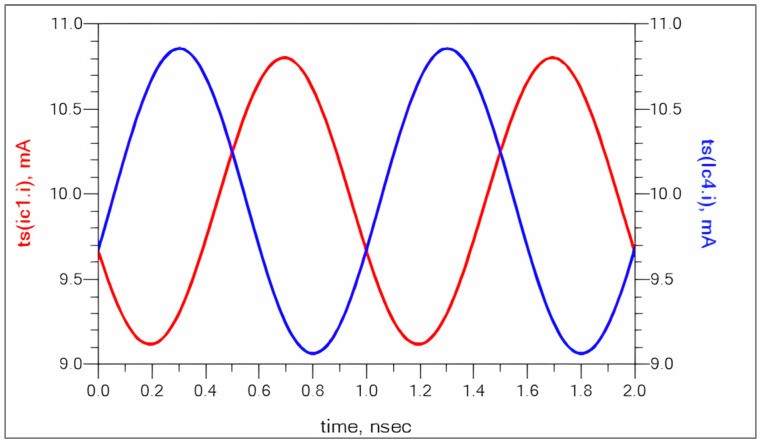
Collector current of RF stage (Q1 and Q4).

Figure 4 shows the time domain collector current waveforms of RF stage transistors. Since the device operating point is around 1.7 V, 10 mA of bias current, the collector current is swinging from 10.8 mA to 9.2 mA.

The gain of the second RF stage is *g*_*m*_ times higher than the first RF stage. Balanced differential signal pairs carry signals of equal amplitude but with a 180° phase shift. If not properly balanced, the differential signaling becomes a source of electromagnetic interference or cross-talk [13], appearing as common-mode noise or voltage at the IF port.

**Figure 5:**
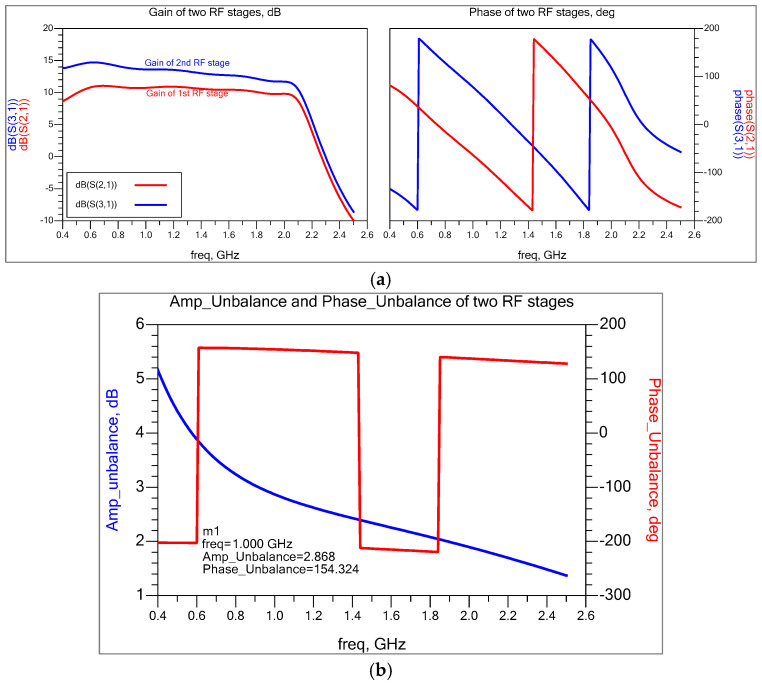
(**a**) Gain and phase of two RF stages, and (**b**) amplitude and phase difference between two RF stages.

Therefore, in order to achieve a close match to the first stage, the gain at the second RF stage must be reduced. This can be done by an emitter degenerating the second stage. Both RF stages are emitter degenerated with chosen impedance, improving input stage linearity and providing noise match. Figure 5a shows the gain and phase of two RF stages; the gain of the second stage is 3 dB higher than the first stage, and the phase difference between the two stages is roughly 154° at 1 GHz (Figure 5b).

A 180-degree phase shift achieves optimal symmetry cancellation, resulting in much improved isolation. However, in practice, it is unusual. As a result, RF-to-IF isolation is inferior to the LO-to-IF and LO-to-RF stages. The RF amplitude difference between the two RF stages is negligible, as balanced symmetry is largely generated from the LO stages, although it may have a slightly deteriorated excellent noise figure.

### 3.2. Local Oscillator: Square Wave Characteristics Generation

**Figure 6:**
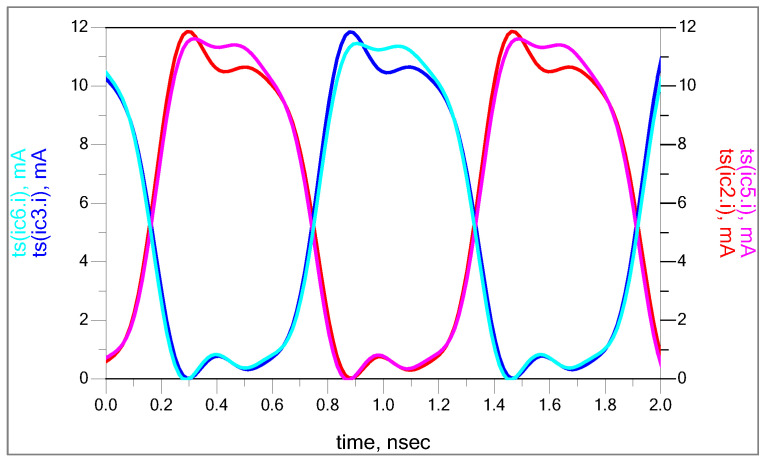
Collector current waveform of LO differential pair.

In the ideal case, we assume the LO differential pair operates in a saturation region with square wave characteristics. At the collector current output, we force the sine wave to look like a square wave, ensuring that the differential pair operates near a saturation region. The waveforms of the upper transistors’ collector currents (Q2 and Q3, and Q5 and Q6 represented in Figure 2) are represented in Figure 6. We lose the device symmetry in the collector current at higher frequencies because of the phase shift of the transconductance $Y_{21}$; the collector current waveforms at the lower side show ringing and limited voltage, while the upper side shows saturation.

The double-balanced Gilbert-cell mixer, reported in Figure 2, consists of two identical single-balance mixers. Therefore, the circuit can be split in two for analytical reasons. Switching cores Q2 and Q3, and Q1 at the RF stage, are present in the first half circuit. Q5 and Q6 are the LO switching cores and Q4 at the RF stage is part of the second half of the circuit.

## 4. Simulation and Experimental Results

The voltage conversion gain of the double-balanced down-conversion mixer can be described as the ratio of the amplitude of the output to that of the input:

(8) $$A_{v}=\frac{2}{\pi }g_{m}R_{L}=\frac{2}{\pi }\frac{I_{tail}}{2V_{T}}R_{L}$$

### 4.1. Simulation and Calculation of Double-Balance Mixer Conversion Gain

For the emitter degeneration case, effective $g_{m}$ can be written as $g_{m\_eff}=\frac{g_{m}}{1+g_{m}Z_{E}}$ while emitter degenerating impedance $Z_{E}$ can be found using $Z_{E}\approx {SL}_{E}+{(R}_{E}\left|\left|\frac{1}{{SC}_{E}}\right)\right)).$ The calculated value of $Z_{E}\;is\approx 21.983+j19.577$ and $of\;\left|Z_{E}\right|\;\approx 29.436$ ohm. The simulation of the circuit is carried out in PathWave Keysight ADS 2023. The layout of the measured prototype was simulated using 3D Keysight ADS 2023 Momentum tools, and simulation measurements were done using the EMcosimulation test setup method.

An LC-tuned circuit load is connected between two outputs at a constant IF of 145 MHz, and each output will have about half of the load connected to the ground. The following formulas can be used to determine the total load impedance: $Z_{L}=(SL||\frac{1}{SC})$ and ${re\{Z}_{L}\}=\sqrt{\frac{L}{C}}\approx 503.94\;\mathrm{ohm}$.

**Figure 7:**
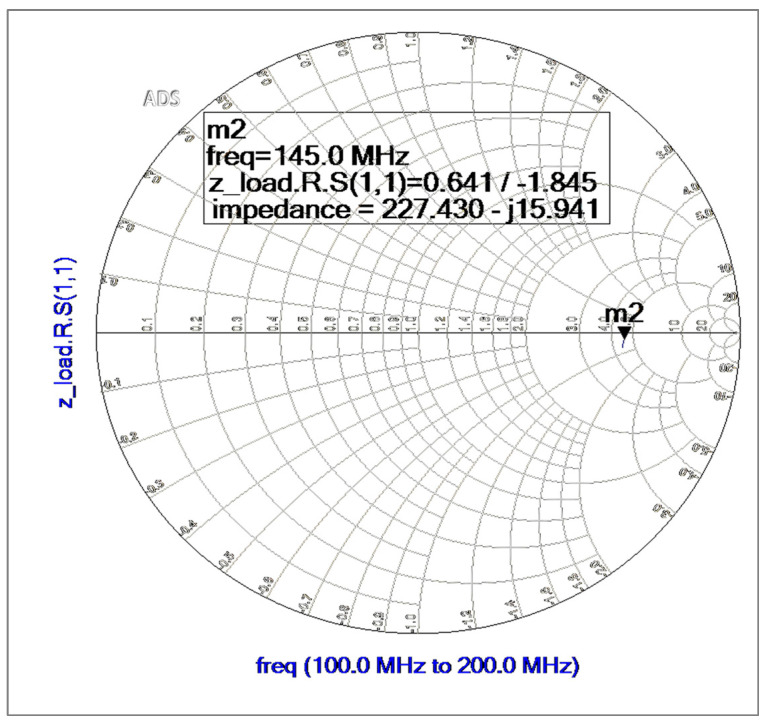
Simulated load impedance at 145 MHz of IF frequency.

Figure 7 shows the simulated load presented at the IF port without matching the 50-ohm system. If we only consider the real part of the load impedance, then $R_{L}\approx 227\;\mathrm{ohm}$. This will approximate the mixer’s conversion gain at the IF port.

**Figure 8:**
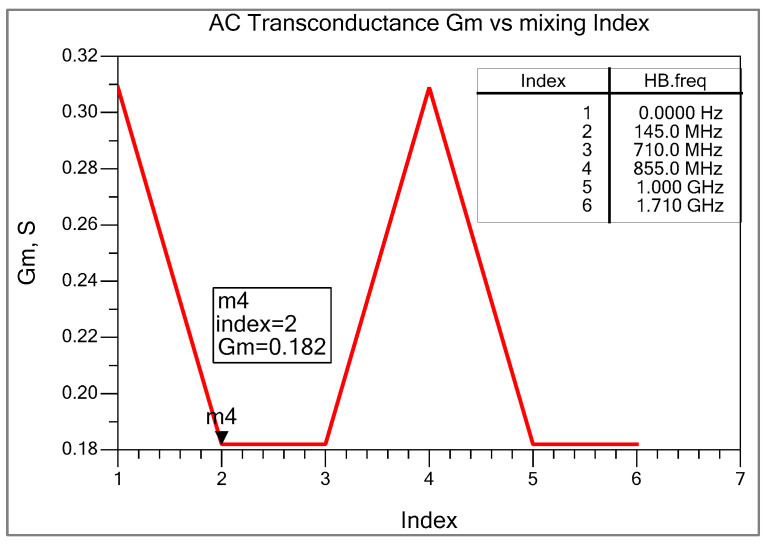
AC transconductance of BJT mixer.

**Figure 9:**
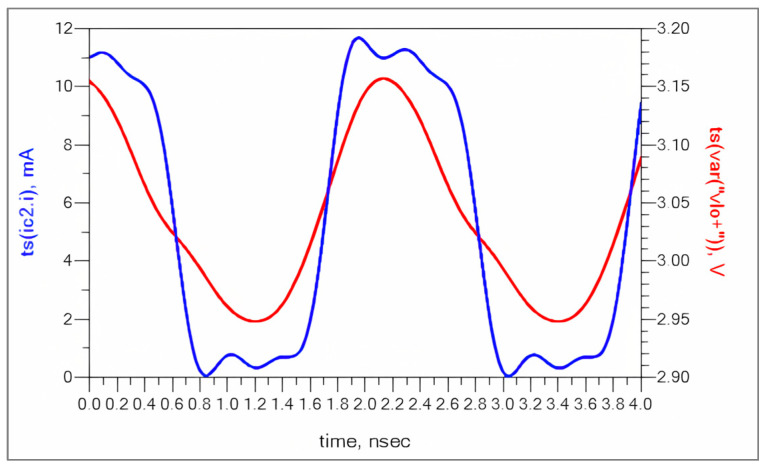
Voltage and current waveform of Q2.

Figure 8 shows the simulated AC transconductance $g_{m}$ of the double-balanced differential mixer. At the same time, the table shown in Figure 8 represents a mixing index of RF and LO signals. The IF frequency of 145 MHz is at index 2. The value of transconductance is about 0.182 S. It can also be calculated from the voltage and current waveform of either Q2 or Q3. The transconductance of the BJT device is shown in Figure 9, and the value can be calculated using $\frac{∆I_{c}}{{∆V}_{be}}$, which is also frequency dependent. In our case, it is 0.189 S.

Substituting the value of $g_{m\_eff}$ into Expression (8) yields the following:

Voltage conversion gain $A_{v}=\frac{2}{\pi }g_{m\_eff}R_{L}=\frac{2}{\pi }\frac{g_{m}}{1+g_{m}R_{E}}R_{L}=\frac{2}{\pi }\frac{0.182*227}{1+(0.182*29.436)}\approx 12.338\;dB$.

**Figure 10:**
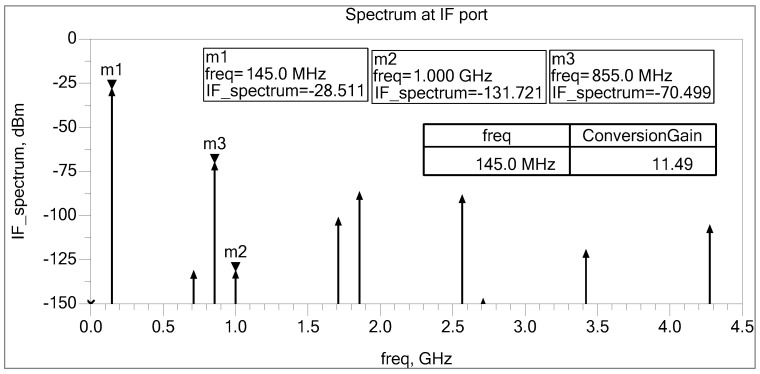
IF spectrum at 145 MHz.

The RF frequency is 1 GHz at −40 dBm, while the LO is 855 MHz at −5 dBm. The IF frequency is down-converted to 145 MHz. Figure 10 shows a simulated frequency spectrum at the IF port. It demonstrates that the LO and RF signals are substantially attenuated, and the simulated conversion gain is around 11.49 dB.

### 4.2. Double-Balanced Down-Conversion Mixer Noise Calculation and Simulation

The noise sources in linear circuits are typically considered to be stationary, but in mixers, the generated noise changes periodically over time, resulting in cyclo-stationary noise processes. This occurs when the dominant noise sources are switched back and forth by the local oscillator [1,16,17,18]. Because of its nonlinear nature, calculating noise requires a nonlinear summation of noise from the RF and LO ports to determine the noise power at the IF. A simulation-assisted approach for these computations that does not rely on harmonic balance techniques has been described in the literature [19,20,21].

Finding the noise-generating mechanism in a single-balanced mixer and doubling the contribution to the IF port are prerequisites for comprehending noise in a double-balanced differential mixer. The analysis will be much easier compared to a Gilbert cell, but it will still offer valuable information on the noise performance of the active mixer. Throughout these calculations, it will be assumed that the output is always taken differentially. The summary shows the main causes of noise and a simple approach for computing the spectral densities of this noise in a single-balanced mixer. The major mechanisms contributing to noise are as follows:

- Once the switching transistors (Q2 and Q3) are fully switched, any noise in the signal path, such as noise from the RF input stage, is manifested at the output as differential noise. This occurs because only one of the outputs is active at a time, and the other switching transistor has a lower impact on the noise.
- When the states of Q2 and Q3 switches change, the noise from the RF input stage Q1 is observed as a common mode and does not add any contribution to the differential output current. However, the switch devices share the DC current and introduce differential noise to the output. In the current commuting mixer, the duration of this period is determined by the magnitude of the LO and decreases with a stronger LO drive.
- Furthermore, significant noise may be present in the LO signal during the switch phase, which could have a similar impact on the output as the noise produced by Q2 and Q3.
- The current-to-voltage stage output consistently introduces differential noise. When a resistive load is used to implement this stage, it typically adds thermal noise.

The noise figure is obtained by combining linear sources and linear sources that have been influenced by the periodic and time-varying characteristics of the circuit [22,23,24]. The detailed analysis is given in [3].

#### 4.2.1. Noise Due to Transconductor Stages

**Figure 11:**
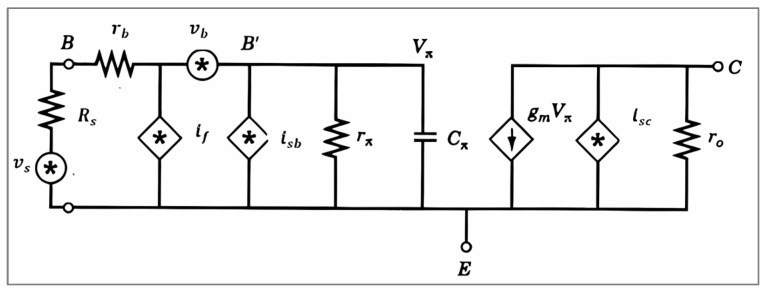
Noise model of the transconductance stage [25].

Figure 11 shows the noise model of the RF transconductance stage. From that, the equivalent noise at the input is as follows:

(9) $$\bar{v_{ni}^{2}}=\frac{1}{{\left(gain\right)}^{2}}\cdot \bar{i_{no}^{2}}=\frac{1}{\frac{g_{m}^{2}z_{\pi }^{2}}{{\left(r_{b}+R_{s}+z_{\pi }\right)}^{2}}}\cdot \left[i_{sc}^{2}+g_{m}^{2}v_{\pi }^{2}\right]$$

In simplified terms, we obtain the following:

(10) $$\bar{V_{n}^{2}}=4KTr_{b}+\frac{2qI_{c}}{g_{m}^{2}}=4KT\left(r_{b}+\frac{1}{2g_{m}}+\frac{g_{m}}{2\beta }\right)$$

(11) $$\bar{I_{n}^{2}}=2qI_{B}=4KT\left(\frac{g_{m}}{2\beta }\right)$$

1. 1. Flicker noise 1f of the transconductor:

In the baseband, 1/f noise is present, but it does not occur at the frequency of the RF signal. Thus, it gets up-converted to the LO frequency and the odd harmonics of the LO signal. Ideally, the flicker noise of the transconductor transistor is shifted out of the baseband, and this should not impact the SNR at the mixing output [1,18].

2. White Noise of Transconductor

**Figure 12:**
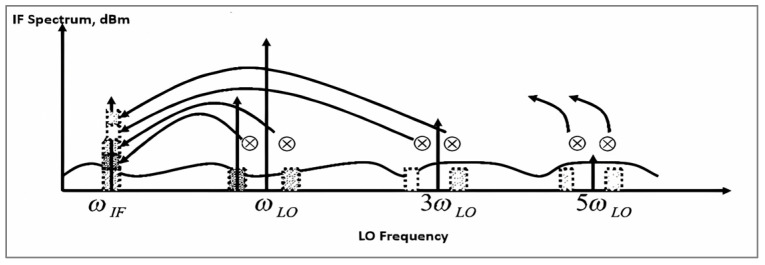
The noise spectral content of the LO frequency and its harmonics down-converted to an IF port in the single-balanced mixer [26].

Figure 12 shows the spectral content of several signals in the single-balanced mixer. The signal resulting from LO mixing comprises odd harmonics of the fundamental frequency $f_{LO}$. The output current of the RF stage at small-signal levels exhibits noise. We assume the power spectral density of noise from the transconductance stage is flat with frequency, i.e., white noise—associated with the transconductor stage [1,3,18].

Hence, mixer communication is considered to be similar to a square wave, with the LO frequency and its odd harmonics converting the corresponding parts of the white noise to the IF [1,18]. This is expressed as follows:

(12) $$\bar{V_{n,o\_RF}^{2}}=n.{\left(\frac{2g_{m1}R_{L}}{\pi }\right)}^{2}\left(4KT\left(r_{b1}+\frac{1}{2g_{m1}}\right)\right)$$

And factor n from [1] is as follows:

(13) $$n=2\left(1+\frac{1}{3^{2}}+\frac{1}{5^{2}}+\cdots \right)=\frac{\pi^{2}}{4}$$

The double-sideband mixer would contribute half of the value indicated in Equation (13) due to the absence of an image sideband. Examining the sidebands of as many harmonics as needed in a mixer circuit is necessary for analyzing the frequency-dependent noise in the RF stage. Calculating the power spectral density is challenging because of inadequate parasitic modeling at very high frequencies.

#### 4.2.2. Noise Due to LO Switching Pair

The local oscillator switching causes the Mixer noise, which is classified as direct switching noise and indirect switching noise.

(i). Direct Switching Noise

**Figure 13:**
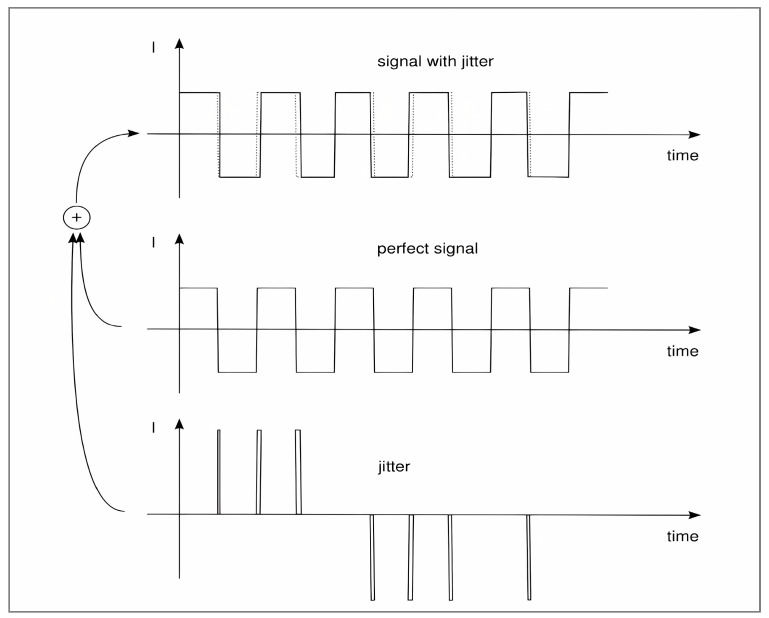
Direct switching noise: The LO signal with jitter can be understood as an undisturbed signal superimposed with a pulse train [18].

**Figure 14:**
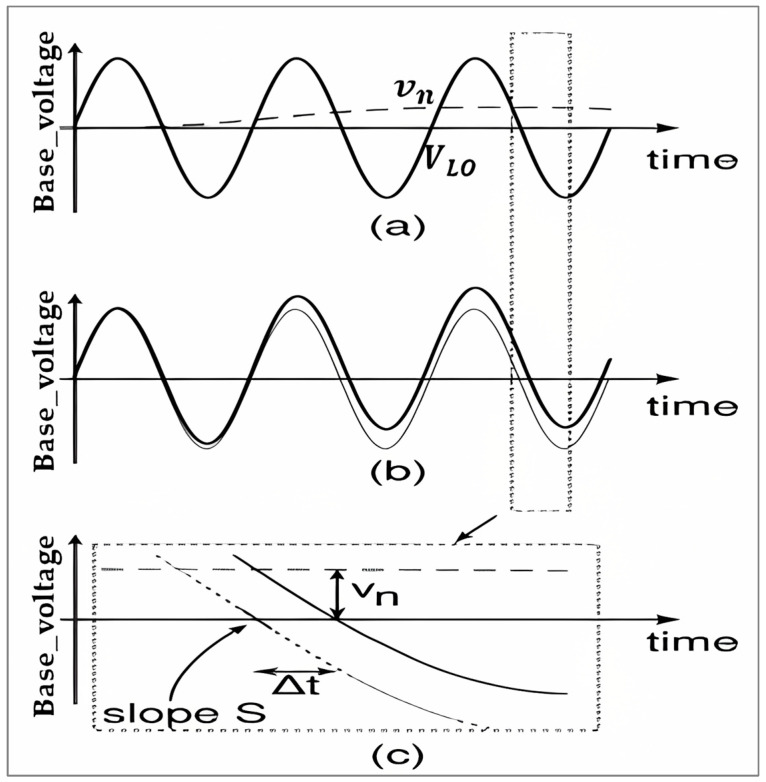
(**a**) Ideal LO base voltage (solid line) and 1/f noise voltage (dashed line); (**b**) actual (solid line) and ideal (dotted line) LO base voltage; and (**c**) the time-offset (jitter) at a zero crossing, calculated from the noise voltage and the slope of the LO signal [18].

Additional 1/f noise, a weak signal overlapped on the LO switching signal, is experienced by the base. Significant changes in the LO signal cause the switching events to occur either forward or backward in time. As a result, the noise causes jitter. Understanding the difference between the switched current with and without jitter is necessary in order to quantify the noise contribution, as illustrated in Figure 13. Subtracting the two signals results in a sequence of pulses with the following properties:

- The pulses are located at the edges of switching times.
- The amplitude can be positive or negative, equal to twice the switched current.
- The pulse width $∆t=\frac{V_{n}(t)}{S}$, where $V_{n}(t)$ is the magnitude of the base noise voltage at the time of switching and S is the slope of the LO voltage applied to the base. Figure 14 shows the relationship.

The sampling frequency is double the LO frequency due to jitter happening at each switching moment or twice during each LO period (a pulse rate of pulse train 2$f_{LO}$). Consequently, the normalized jitter noise current for one period is as follows:

(14) $$I_{o,n}=\frac{2}{T_{LO}}.2I_{RF}.\frac{V_{n}}{S}$$

The slope (S) of the sinusoidal LO signal can be given as follows:

For a differential LO signal, $V_{Lo}d=2{sin\omega }_{LO}t$; therefore, $S=\frac{dV_{LO}d}{dt}=2V_{LO}\omega_{LO}$. So, $ST_{LO}$ will be ${4\pi V}_{LO}$. The baseband noise spectrum becomes the following:

(15) $$I_{o,n}(f)=\frac{1}{{\pi V}_{LO}}.I_{RF}.\;V_{n}(f)$$

The noise $V_{n}(t)$ from the LO switch is only transferred to the output during the zero crossing. Both switches contribute noise to the mixer output when they are on; no noise is contributed when one switch is off, and when the other switch is on, it also contributes no noise due to its functioning as a cascade structure with a fixed tail current of $I_{RF}$ by the RF input transconductance stage. The noise current at the mixer output is a series of pulses occurring at twice the LO frequency rate, with a height equal to $2I_{RF}/S$, and a width that is randomly modulated by noise. The input noise is stationary and white, and its power spectral density is given by the following:

(16) $$\bar{V_{n,s}^{2}}=4KT\left(r_{b2}+\frac{1}{2g_{m2}}\right)$$

(17) $${{[g}_{m2}]\;}_{zerocrossing}=\frac{2I_{RF}}{∆V}$$

where $g_{m2}$ is the switch transconductance at zero crossing.

The power spectral density of the output noise current is given by

(18) $$\bar{I_{n0,s}^{2}}=\int_{o}^{T_{LO}}p^{2}\left(t\right)\bar{V_{n,s}^{2}}=\left(\frac{2}{T_{LO}}\right){\left(\frac{2I_{RF}}{S}\right)}^{2}\left(\frac{1}{T_{S}}\right)\bar{V_{n,s}^{2}}$$

(19) $$=\left(\frac{4I_{RF}}{S\cdot T_{LO}}\right)\left(\frac{2I_{RF}}{S\cdot T_{S}}\right)4KT\left(r_{b2}+\frac{1}{2g_{m2}}\right)$$

Substituting the value of $S\cdot T_{LO}$ = ${4\pi V}_{LO}$, we obtain the following:

(20) $$\bar{I_{n0,s}^{2}}=\left(\frac{I_{RF}}{{\pi V}_{LO}}\right)2KT\left(2r_{b2}g_{m2}+1\right)$$

The output power spectral density depends on LO magnitude ($V_{LO}$) and bias current ($I_{RF}$).

(ii). Indirect Switching Noise

Indirect switching noise depends on the LO frequency and BJT capacitances existing at the virtual ground. The noise voltage $v_{n}$ << LO signal, so it can be treated as a switched voltage of slowly varying amplitude. The switching edges will be a time constant, enabling a current through the capacitance $C_{p}$ associated with transconductance stage Q1:

(21) $$C_{p}=C_{be2}+C_{be3}+C_{js1}+C_{bc1}$$

(22) In,o=2TLO∫0TLO2Cpddtvp(t)

This leads to the noise contribution:

(23) $$I_{n,o}=\frac{2}{T_{LO}}C_{p}v_{n}=2f_{LO}C_{p}v_{n}$$

In conclusion, indirect switching noise is much smaller than direct switching noise.

#### 4.2.3. Noise Due to Load

Noise due to load $R_{L}$ is $4KTR_{L}$ and the output is differential; total thermal noise down-converted to IF will be as follows:

(24) $$\bar{V_{n,R_{L}}^{2}}=8KTR_{L}.$$

#### 4.2.4. Total Mixer Output Noise

From Equations (12), (20), and (24), the total noise at mixer output can be written as follows:

(25) $$\bar{V_{n0,total}^{2}}=\bar{V_{n,R_{L}}^{2}}+2(\bar{I_{n0,s}^{2}})\;{R_{L}}^{2}+\bar{V_{n,o\_RF}^{2}}$$

(26) $$=8KTR_{L}+\left(\frac{I_{RF}}{{\pi V}_{LO}}\right)4KT\left(2r_{b2}g_{m2}+1\right){R_{L}}^{2}\;\;\;\;\;\;\;\;\;\;\;\;\;+n.{\left(\frac{2g_{m1}R_{L}}{\pi }\right)}^{2}\left(4KT\left(r_{b1}+\frac{1}{2g_{m1}}\right)\right)$$

The simplified expression for signal-balanced mixer noise down-converted to IF is given by the following:

(27) $$\bar{V_{n0,total}^{2}}={4KTR_{L}}^{2}\left[\frac{2}{R_{L}}+\left(2r_{b2}g_{m2}+1\right)\left(\frac{I_{RF}}{{\pi V}_{LO}}\right)+{g_{m1}}^{2}\left(r_{b1}+\frac{1}{2g_{m1}}\right)\right]$$

#### 4.2.5. Double-Balanced Mixer Noise Figure Prediction Calculation and Simulation Validation

Due to the double transconductance stages and switches in the double-balanced mixer, the noise contribution down-converted to the IF port is doubled. Therefore, the total noise at the mixer output can be written as from Equation (27):

(28) $$\bar{V_{n0,total}^{2}}={4KTR_{L}}^{2}\left[\frac{2}{R_{L}}+2\left(\left(2r_{b2}g_{m}+1\right)\left(\frac{I_{RF}}{{\pi V}_{LO}}\right)\right)+2\left({g_{m}}^{2}\left(r_{b1}+\frac{1}{2g_{m}}\right)\right)\right]$$

The input referred noise of the given mixer is as follows:

(29) $$\bar{V_{ni,total}^{2}}=\frac{1}{{\left(gain\right)}^{2}}\bar{V_{n0,total}^{2}}$$

(30) $$\bar{V_{ni,total}^{2}}=\frac{{4KTR_{L}}^{2}\left[\frac{2}{R_{L}}+2\left(\left(2r_{b2}g_{m}+1\right)\left(\frac{I_{RF}}{{\pi V}_{LO}}\right)\right)+2\left({g_{m}}^{2}\left(r_{b1}+\frac{1}{2g_{m}}\right)\right)\right]}{{\left(\frac{2g_{m1}R_{L}}{\pi }\right)}^{2}}$$

(31) $$\bar{V_{ni,total}^{2}}=\pi^{2}KT\left[\frac{2}{{{g_{m}}^{2}R}_{L}}+2\left(r_{b1}+\frac{1}{2g_{m}}\right)+\left(\frac{2}{{g_{m}}^{2}}\left(2r_{b2}g_{m}+1\right)\left(\frac{I_{RF}}{{\pi V}_{LO}}\right)\right)\right]$$

So, the single-sideband noise figure can be given as follows:

(32) $${NF}_{SSB}=1+\frac{\bar{V_{ni,total}^{2}}}{4KTR_{S}}$$

The noise equation derived in (28) and (31) for output- and input-referred noise assumes that RF stages do not feature emitter degeneration. When the emitter is degenerated, ${\;g}_{m}$ is substituted with ${\;g}_{m_{eff}}$ as derived in Section 3.1. In the following, we will estimate the noise figure of the double-balanced mixer illustrated in Figure 2.

The internal base resistances of BFP840 ($r_{b1}$ at RF stage) and BFP640 ($r_{b2}$ at LO stage) were found in the spice parameter file: the values are 16.87 ohms and 4.406 ohms, respectively. The simulated transconductance $g_{m}$ is approximately 0.182 S. The local oscillator port has a power of −5 dBm; according to the simulation, this represents about 355 mVp-p when converted to mV for the 50-ohm system.

Upon calculating the individual terms listed in Equation (31), we obtain the data from Table 1:

Substituting all of the values in Equation (31) and then in (32), the result of $\frac{\bar{V_{ni,total}^{2}}}{4KTR_{S}}$ will be roughly 5.264251.

$${NF}_{SSB}=10log\left(1+5.264251\right)=10\log\left(6.264251\right)=7.968692\;\mathrm{dB}.$$

**Figure 15:**
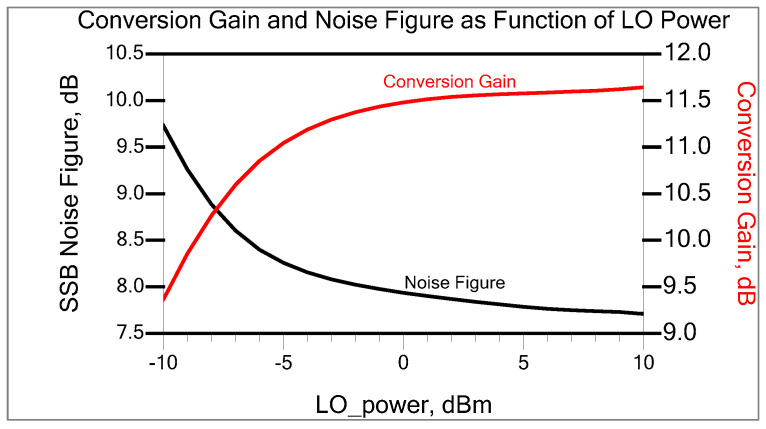
Conversion gain and SSB noise figure as function of LO power.

The optimal condition for the gain and noise figure as a function of the LO drive is displayed in Figure 15. The predicted approximation is about 7.9686 dB, while the simulated noise figure measures about 8.31 dB when the LO power is at −5 dBm.

**Figure 16:**
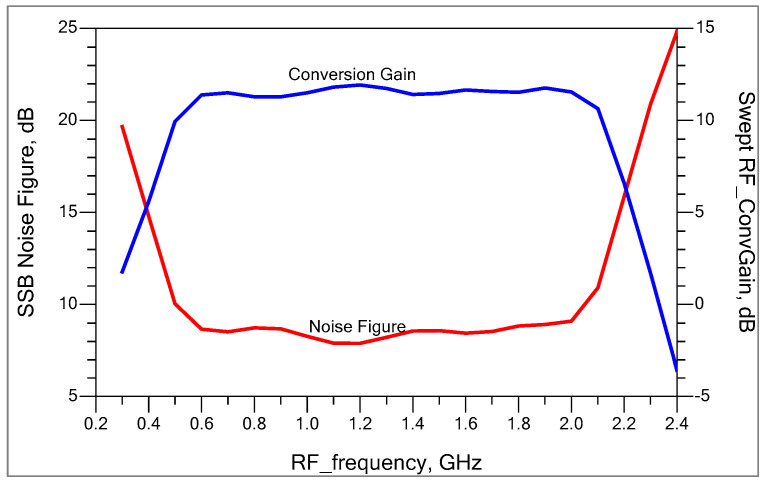
Single-sideband noise figure and conversion gain versus RF input frequency for fixed IF at 145 MHz.

We swept the RF and LO frequency across the range of 400 MHz to 2.5 GHz while maintaining a constant IF of 145 MHz in order to determine the operating bandwidth of the down-conversion mixer. Figure 16 shows the single-sideband noise figure and simulated conversion gain as a function of RF frequency.

We simplified the gain and noise figure calculation by neglecting the internal capacitances of the transconductance and switching stages, since RF and LO frequencies are much lower than $f_{T}$. The assumption is that the $C_{\pi }$ and $C_{\mu }$ of each device are small enough to act as open circuits at all signal frequencies of interest. Figure 16 shows that the gain stays nearly constant (11 dB +/− 0.5 dB) between 600 MHz and 2000 MHz.

### 4.3. Linearity

The performance of most communication systems is limited by the mixer’s dynamic range. The linearity of the mixer relies on the LO switches, the RF transconductance stages, and the tail current source. If the LO switches are ideal, the impedance observed when looking into the emitter of Q2 and Q3 is equivalent to that of the common base stage. i.e.$,\;\frac{1}{g_{m2,3}}$. The voltage gain at the collector terminal of Q1 is extremely low, i.e., $\frac{g_{m1}}{\;g_{m2,3}}$, indicating that the ${IIP}_{3}$ of the mixer is determined by the RF transconductance stage Q1 rather than the LO switching core [27,28,29,30].

**Figure 17:**
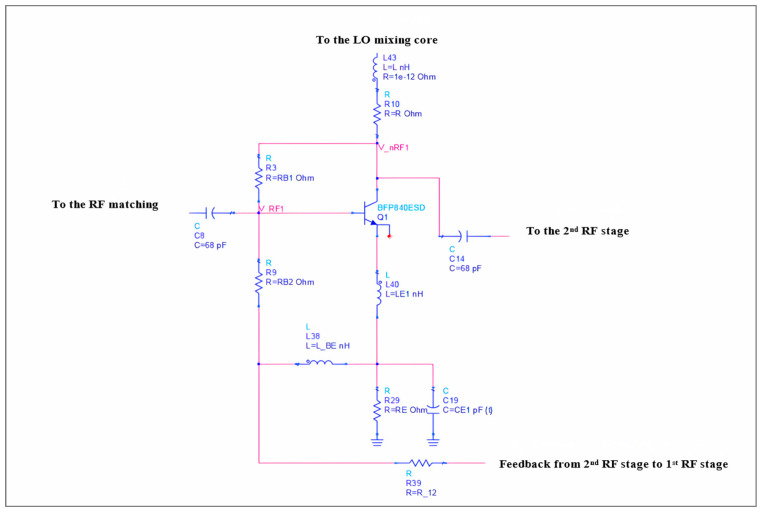
RF stage with feedback network.

The linearization of the RF stage requires only the RF signal to be fed back while minimizing the LO and IF signals at the RF port is necessary to maintain port-to-port isolation. The collector currents of Q2, Q3, Q5, and Q6 are combined to ensure that the RF and LO signals cancel at the IF ports. The feedback applied only serves to linearize the RF stages and does not affect the mixing stages. Simultaneously, a shunt voltage feedback (Figure 17) is established between the collector of Q1 and the RF signal port node ($V_{\_RF1}$, $V_{\_nRF1}$) through the resistor R_B1_.

Figure 17 illustrates that inductive series feedback is located in the tail of transistors Q1 and Q4. The degenerative impedance of the first RF stage is determined by a 4.7 nH inductor ($Z_{E1}\approx 21.983+j19.577$), while a 2.2 nH inductor ($Z_{E2}\approx 25.378+j12.609$) was sufficient for linearization in the second stage. The current gain β varies by approximately 10 percent from one device to another. The variation in degenerative impedance in the two RF stages is much smaller compared to the β variation. The chosen inductor values are carefully designed and optimized to provide noise match.

The input impedance $Z_{in}$ of the dual feedback double-balanced mixer at RF is set by the feedback element R_B1_ and degenerative impedance $Z_{E}$:

(33) $$re\left\{Z_{in}\right\}\approx \;R_{B1}||(r_{b}+\frac{\beta }{g_{m}}+Z_{E})$$

The analysis is a large signal and is based on the Ebers–Moll equation. The derivation of the output current $I_{F1}$ at the IF port can be written as follows:

(34) $$I_{c2}+I_{c6}=I_{c1}I_{c2}+I_{c4}I_{c6}$$

where

(35) $$I_{c1}=\frac{\alpha_{F}\sqrt{I_{bias}}}{\left(e^{\frac{-\left(\left(V_{{RF}^{+}}\right)-\left(V_{{RF}^{-}}\right)\right)}{V_{T}}}+1\right)}\;and\;I_{c4}=\frac{\alpha_{F}\sqrt{I_{bias}}}{\left(e^{\frac{\left(\left(V_{{RF}^{+}}\right)-\left(V_{{RF}^{-}}\right)\right)}{V_{T}}}+1\right)}$$

(36) $${\;I}_{c2}=\frac{\alpha_{F}\sqrt{I_{bias}}}{\left(e^{\frac{-\left(\left(V_{{LO}^{+}}\right)-\left(V_{{LO}^{-}}\right)\right)}{V_{T}}}+1\right)}\;and\;{\;I}_{c6}=\frac{\alpha_{F}\sqrt{I_{bias}}}{\left(e^{\frac{\left(\left(V_{{Lo}^{+}}\right)-\left(V_{{Lo}^{-}}\right)\right)}{V_{T}}}+1\right)}$$

From this, the output voltage is simply the following:

(37) $$V_{IF1}=(I_{c1}I_{c2}+I_{c4}I_{c6})Z_{L}$$

The feedback current $I_{f}$ due to $R_{B1}$ is as follows:

(38) $$I_{f}=\frac{V_{\_nRF1}-V_{\_RF1}}{R_{B1}}$$

The voltage at node $V_{\_nRF1}$ from Figure 17 can be written as follows:

(39) $$V_{\_nRF1}=({\;I}_{c2}+{\;I}_{c3})(R+sL)$$

A detailed analysis of the feedback current $I_{f}$ is given in [3]; from that feedback, current $I_{f}$ is as follows:

(40) $$I_{f}=\frac{\left(\left(\alpha_{F}\cdot \frac{\alpha_{F}\cdot I_{bias}}{\left(e^{\frac{-\left(\left(V_{{RF}^{+}}\right)-\left(V_{{RF}^{-}}\right)\right)}{V_{T}}}+1\right)}\;\right)(R+sL)-V_{\_RF1}\right)}{R_{B1}}$$

The $V_{\_RF1}$ term consists of the RF signal frequency, while $V_{\_nRF1}$ consists of the LO and IF frequency signal components. No exponential terms related to the LO or IF signal components are present in the feedback signal, resulting in their cancellation. Since there is only one term proportional to the RF signal in Equation (40), this leads to a reduction in the input signal magnitude and linearization of the mixer.

**Figure 18:**
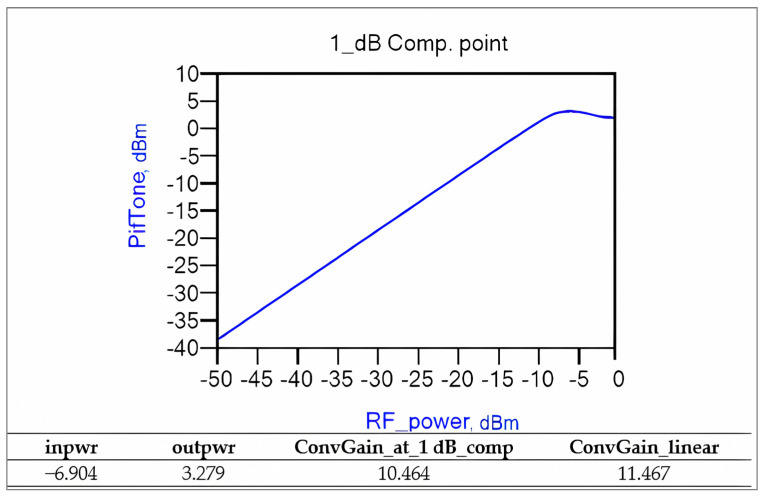
Compression characteristics of the down-conversion double-balanced mixer.

The graph in Figure 18 depicts the compression behavior of the down-conversion mixer relative to the RF input power. In this scenario, the frequency of the local oscillator is 855 MHz, the RF frequency is 1000 MHz, and the RF power increases in every simulation. The uncompressed gain measures approximately 11.4 dB, which is slightly lower than our initial estimate, and it decreases by 1 dB at −6.9 dBm (approximately 143 mV). Accurately measuring the 145 MHz IF amplitude in the time domain is challenging due to the presence of LO feedthrough and signal distortion. Therefore, the analysis focuses on the fast Fourier transform (FFT) of the input and output [30,31,32,33,34,35,36].

**Figure 19:**
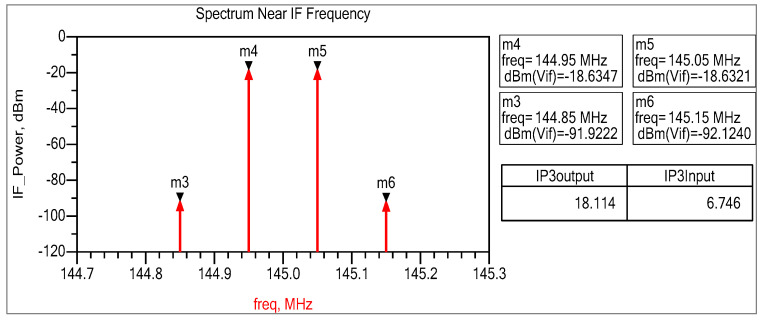
Two-tone test of a 1GHz down-conversion double-balanced mixer.

**Figure 20:**
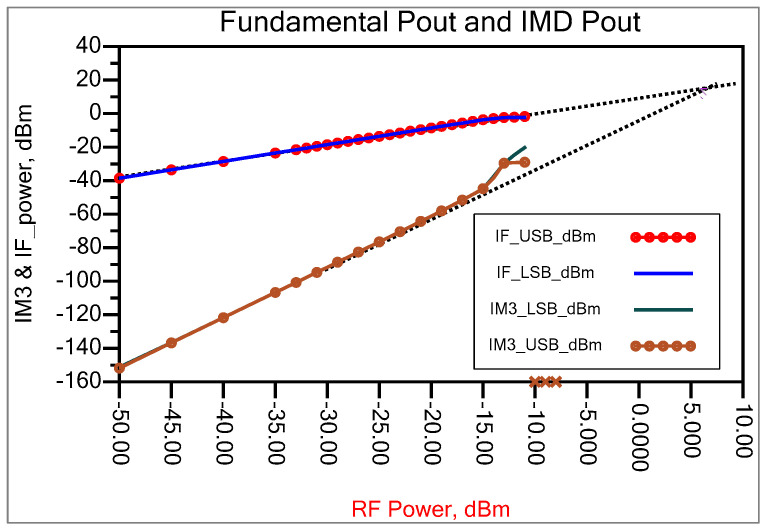
Extrapolation of IP3 from fundamental and third harmonic tone power.

To evaluate the input IP3 of the mixer, we introduce two sinusoidal signals to the input, each with frequencies of 999.95 MHz and 1000.05 MHz. The peak amplitude of both signals is set at −30 dBm. If the signal amplitude is too low, the ${IM}_{3}$ components are affected by the FFT noise floor; if it is too high, the circuit may encounter higher-order nonlinearities. Figure 19 shows the mixer’s down-converted spectrum, demonstrating a difference of $∆P=P_{out}-P_{IM3}=73.214\;\mathrm{dB}$ between the fundamental and ${IM}_{3}$ tones. After this value is divided by two, and the input tone power is then added, we get ${IIP}_{3}$ = +6.716 dBm in a 50-ohm system. A geometric interpretation of the above relationship is presented in Figure 20.

### 4.4. Mixer Layout Design and EM Simulation

**Figure 21:**
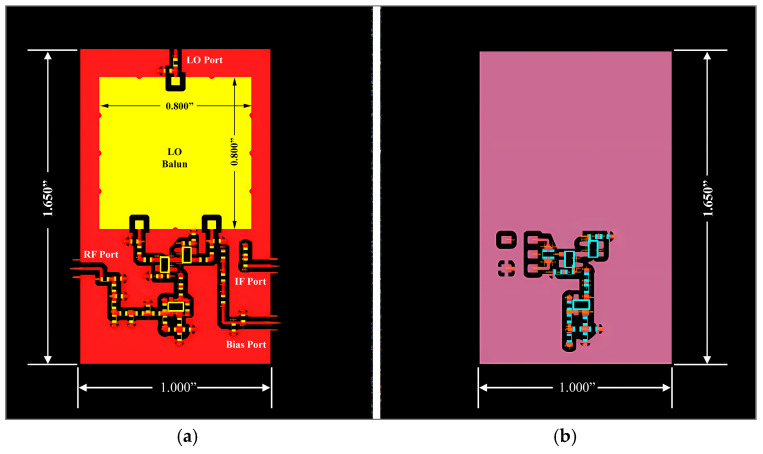
Optimized final EMcosim layout of the novel double-balanced mixer: (**a**) top view of the PCB and (**b**) bottom view of the PCB.

**Figure 22:**
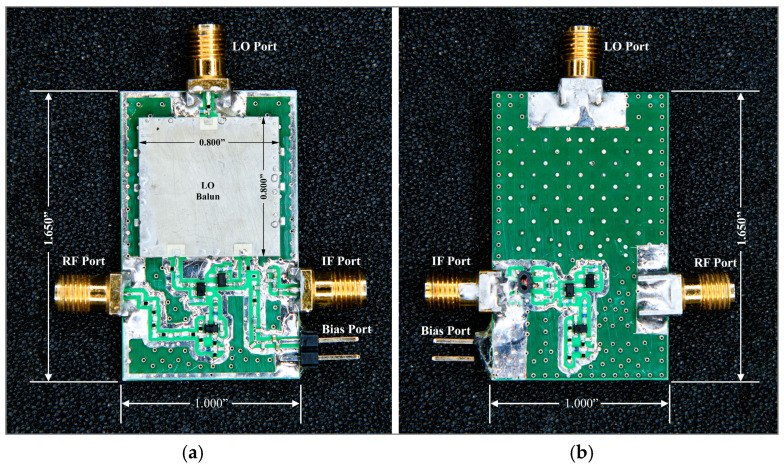
Developed prototype of novel down-conversion double-balanced mixer for validation purposes: (**a**) top view of the PCB and (**b**) bottom view of the PCB.

Figure 21 and Figure 22 present the simulated and, respectively, validated prototype of the down-conversion double-balanced differential mixer.

The planar mixer board measures roughly 1.650’’ × 1.000” × 0.140” overall and includes the local oscillator balun (0.800” × 0.800” × 0.084”). It is fabricated on a six-layer (each layer is Rogers 4003 1 oz Cu 8 mil height substrate) board. The top layer consists of the first half of the circuit’s symmetry and local oscillator balun, while the bottom layer consists of the other half of the circuit’s symmetry and IF transformer. The DC bias line, common ground, and signal connection of the LO and RF signal run in the intermediate layers.

Initially, the two-layer board was used for implementing the mixer design presented in Figure 2. Transistors on the board were not isolated from one another, which caused problems with cross-talk between LO and RF signals. Active devices were arranged symmetrically to address this layout problem (the layout optimization is visible in Figure 21). To reduce the LO–RF feedthrough, the layout minimized cross-overs of the LO and RF signal lines wherever feasible.

### 4.5. Experimental Validation of Down-Conversion Double-Balanced Mixer Prototype

This section will discuss the measured parameters of the improved double-balanced Gilbert-cell mixer.

#### 4.5.1. Gain, Isolation, and Return Loss Measurement

The gain, isolation (LO–RF, LO–IF, and RF–IF), and return loss of the RF port, LO port, and IF port were measured on the Rohde & Schwarz ZNB 20 vector network analyzer mixer mode test setup. The setup was calibrated using the Rohde & Schwarz (Germany) ZV-Z52 calibration unit and Rohde & Schwarz thermal power sensor NRP-Z55 (PUOSM calibration) [37,38].

**Figure 23:**
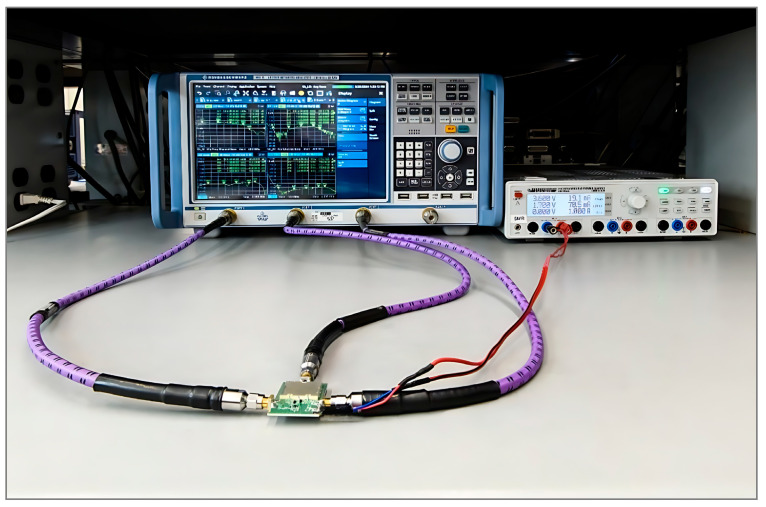
Mixer mode measurement setup. DUT measures conversion gain and port-to-port isolation.

Figure 23 shows the measurement setup where RF and LO frequencies are sweeping from 300 MHz to 2500 MHz with the fixed IF difference of 145 MHz. This test setup is performing down conversion. Since the NRP-Z55 can only measure precise power levels between −30 dBm and +20 dBm, the power level of the RF port was set to −25 dBm; CAD tools, however, do not have that limitation.

**Figure 24:**
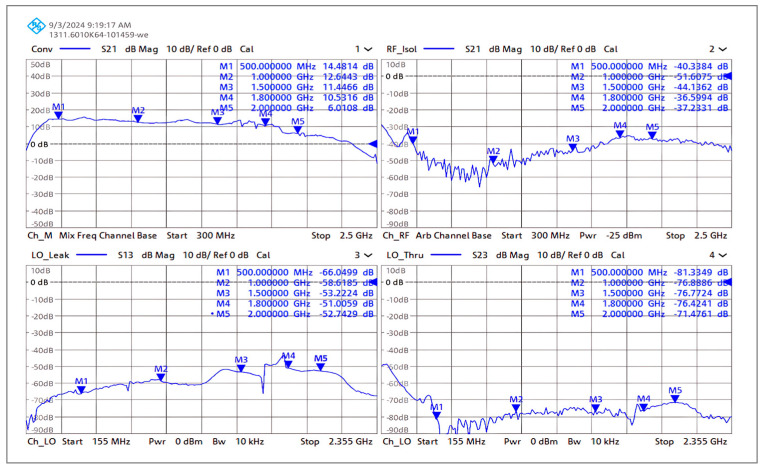
Measured conversion gain and RF–IF (RF isol) isolation of the down-conversion mixer prototype as a function of RF frequency. LO–IF (Lo thru) and LO–RF (Lo leak) isolation are measured as a function of LO frequency. Test conditions: RF frequency: 300 MHz to 2.5 GHz at −25 dBm. LO frequency: 155 MHz to 2.355 GHz at 0 dBm and fixed IF 145 MHz. (Supply voltage of 3.6 V and total current of 19 mA).

The measured conversion gain of the developed down-conversion mixer is illustrated in Figure 24. Since conversion gain is directly correlated with transconductance, it exhibits variation in gain as the transconductance decreases with increasing frequency. It shows that conversion gain varies 12 dB ± 1 dB from 600 MHz to 1.8 GHz. On the other hand, gain falls off above 1.8 GHz as a result of *Cπ*, *Cμ*, and substrate capacitance. The variance in simulated performance can be attributed to the accuracy in determining the parasitic capacitance of the substrate (bond pad) during the simulation. Furthermore, due to the perfect symmetry in the layout, BJT-based Gilbert-cell mixers provide outstanding LO–IF isolation of up to 60 dB compared to conventional active mixers [39].

**Figure 25:**
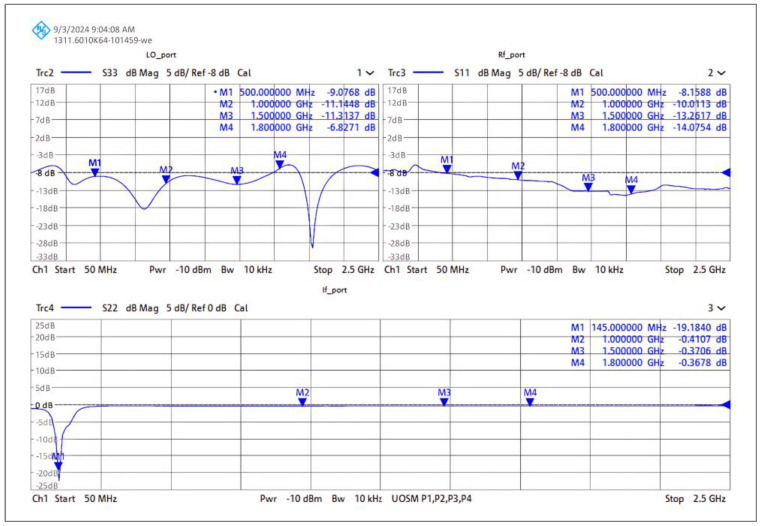
Measured return losses of the down-conversion mixer prototype.

Figure 25 shows the measured return loss of the down-conversion double-balanced mixer. The tuned circuit exhibits the ideal match to the system impedance at around 145.5 MHz because of the component’s standard values and tolerances.

#### 4.5.2. Noise Figure Measurement Using a Y-Factor Measurement Technique

The 145 MHz IF filter selectivity of the IF port ensures that the IF spectrum will not contain any image frequencies. The SSB noise figure was measured using the Rohde & Schwarz FSWP with an EID (Electronic Instrumentation Division P/N 7626-1 frequency 10 MHz–26 GHz) noise source connected to the RF port. The measurement employs fixed LO down conversion since the LO frequency cannot be swept through the internal generator of FSWP. Details of the noise figure measurement for frequency converting DUT are given in the application note of Rohde & Schwarz [40].

**Figure 26:**
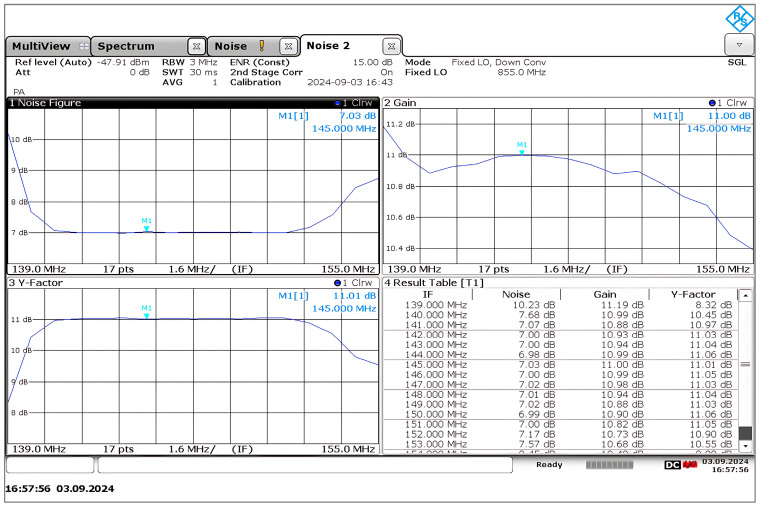
The noise was measured at the IF port using the Y-factor method. Test conditions: RF frequency: 994 MHz to 1010 MHz. Fixed LO frequency: 855 MHz at 0 dBm.

Figure 26 shows the noise figure measured at 145 MHz. Additionally, the graph also indicates that the bandwidth of the tuned filter is roughly 10 MHz, and the noise figure is 7 dB ± 0.1 dB. The gain will reach its peak at the center frequency and then progressively decrease toward the higher and lower frequencies, depending on the Q of the circuit.

As can be seen in Figure 15 and Figure 26, the noise figure that was measured for the mixer is lower than the result that was obtained from the simulation. According to the Friss Formula, the input stage has the most significant impact on the total noise figure, and stages further down in the chain will contribute less to the overall noise figure. The total mixer noise contribution derived in Equation (31) mainly depends on the internal base resistance and transconductance of each transistor. At the RF input stage, the internal base resistance of Q1 and Q4 generates a thermal noise and directly adds to the input-referred noise of the mixer and ultimately reduces the SNR (signal-to-noise ratio) at the input, which leads to having a higher noise figure. Thus, lower internal base resistance will reduce the thermal noise at the input, improving the SNR at the input and resulting in a lower noise figure. However, due to complex modeling and curve-fitting processes, there will be some inadequacies in the extraction of the transistor’s spice parameter. This can be attributed to the fact that the ADS simulator uses the spice parameter library data that Infineon supplied, which may be pessimistic. Inevitably, we rely on the simulator’s predictive accuracy.

#### 4.5.3. Linearity Measurement: IIP3 and 1 dB Compression Point

Both the Rohde & Schwarz FSWP spectrum analyzer and the SMA100A (with a frequency range of 9 KHz to 6 GHz) signal generator were used to measure the 1 dB compression point and IP3 measurement tests.

**Figure 27:**
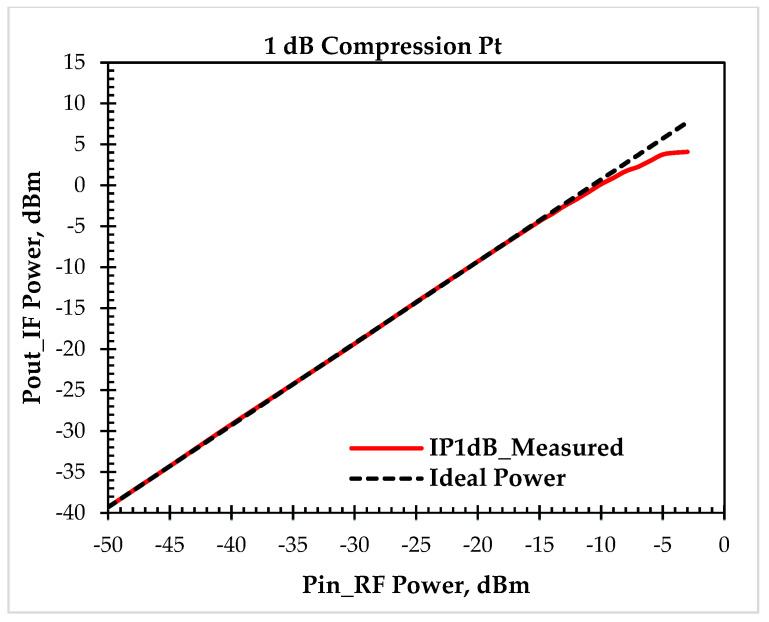
Measured compression characteristic of the down-conversion double-balanced mixer prototype.

Figure 27 illustrates the compression characteristic of the down-conversion double-balanced mixer by showing the IF output as a function of RF frequency. The parameters used for the measurements are $f_{LO}=855\;MHz,and\;f_{RF}=1000\;MHz$ with the RF power being incrementally increased for each data point. When the RF power reaches the value of −8 dBm, the initial gain, which is roughly 10.79 dB without compression, drops by 1 dB.

**Figure 28:**
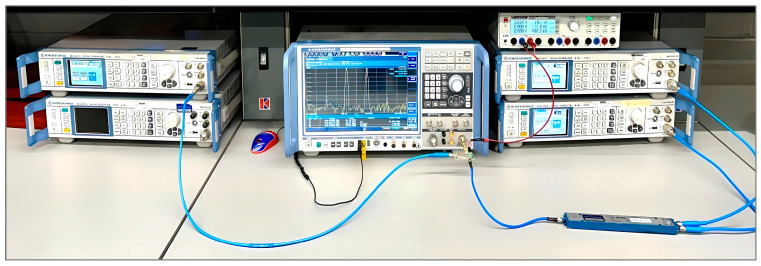
Two-tone test measurement setup, showing DUT is measuring the third-order intermodulation product.

**Figure 29:**
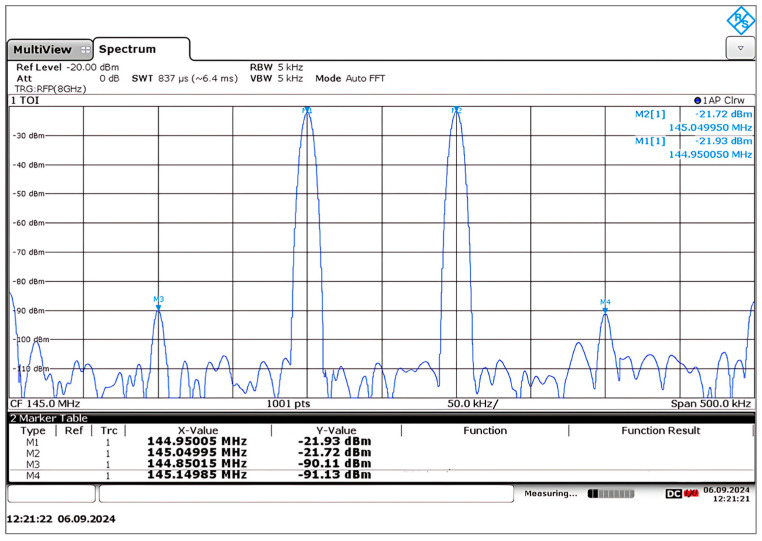
Measured two-tone test of the 1 GHz down-conversion double-balanced mixer.

The input IP3 point of the mixer is measured by applying two sinusoidal tones to the input at frequencies of 1000.05 MHz and 999.95 MHz. Every tone has a peak amplitude of −30 dBm. The two-tone measurement setup used to determine the IIP3 point is presented in Figure 28. A 3 dB power divider coupler separated RF1 and RF2 tones, and two tones were spaced 100 KHz apart, generating two IF peaks at a frequency of 144.95 MHz and 145.05 MHz, while their third-order intermodulation products were generated at 144.85 MHz and 145.15 MHz, as shown in Figure 29. This shows the mixer’s down-converted spectrum, revealing a difference of ∆P=68.18 dB between the fundamental and ${IM}_{3}$ tones, yielding ${IIP}_{3}$ = 4.7 dBm measured in a 50-ohm system. The third-order intercept point can be calculated as follows [41,42]:

(41) $${IIP}_{3}=P_{in}+\frac{∆P}{2}$$

where $P_{in}=-30\;dBm$ and $∆P=P_{out}-P_{IM3}$.

Now, for a lower sideband tone, $∆P=P_{out}-P_{IM3}=-21.93-\left(-90.11\right)=68.18$ and thus, ${IIP}_{3\_LSB}=4.09\;dBm$. For an upper sideband tone, $∆P=P_{out}-P_{IM3}=-21.73-\left(-91.13\right)=68.18$ and thus, ${IIP}_{3\_USB}=4.7\;dBm$. The ${OIP}_{3}$ point is simply ${IIP}_{3}+Gain$, so the calculated ${OIP}_{3}$ point was about 15 dBm.

**Figure 30:**
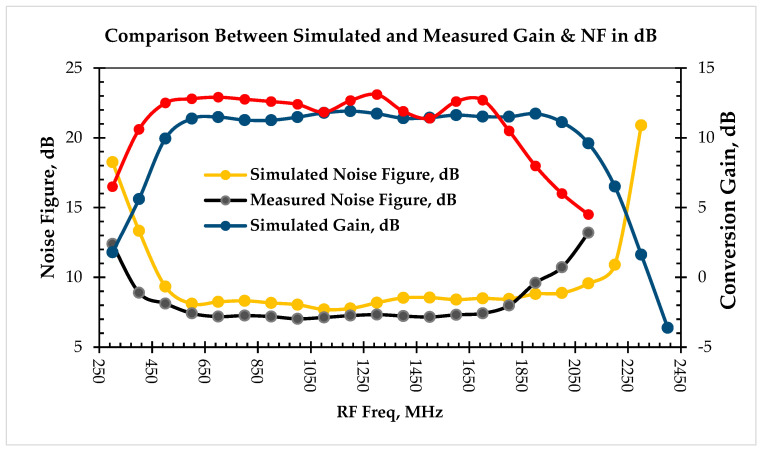
Comparison between simulated and measured gain and noise figure as a function of RF frequency.

Figure 30 compares the noise figure and conversion gain between the measured and simulated values as a function of RF frequency. The IF is down converted to 145 MHz. Therefore, the LO frequency is 145 MHz less in comparison to the RF frequency. The frequency response of the LO balun determines the local oscillator bandwidth. The LO and RF signals are AC coupled through a coupling capacitor (68 pF), which sets the lower frequency limit. There is no accurate model for the parasitics (mainly substrate capacitance) that existed from the board layout; these effects are dominant at higher frequencies. This is the main reason that the measured gain and noise figure bandwidth are less in comparison to the simulated ones.

The noise floor of the circuit is about −166 dBm, and IP3 and the 1 dB compression points are roughly 5 dBm and −8 dBm, respectively, and so the available dynamic range is calculated as follows: (−174 dB–1 dB compression point + NF). From this, (−174 − (−8) + 7 ≈ 159 dBm/Hz) is the available dynamic range (however, Table 2 shows (−174 − (−13) + 18 ≈ 143 dBm/Hz, Ref. [33]).

Table 2 displays a comparison of several performance measures of the proposed double-balanced mixer, integrating the performance improvement approaches in this study with existing ones in the literature (only the most relevant papers are presented in the table; the complete list is [33,34,35,36,37,38,39,40]).

In Table 3, the parametric performance of the down-conversion double-balanced mixer is compared between simulated and measured results.

## 5. Conclusions

This manuscript offers a novel approach to designing a double-balanced mixer without having the need for a bulky transformer at the RF stage, which is suitable for a wide array of applications, including military radar, satellite communication (SATCOM), and cellular base stations.

The down-conversion mixer is made up of a single-ended to a differential-balanced RF stage, a dual feedback linearization for the RF stage, a local oscillator balun, local oscillator mixing cores, and a fixed IF tuned circuit connected between two outputs to serve as a load at 145 MHz. The dual feedback linearization technique improves nonlinear transfer characteristics to increase IIP3 and the 1 dB compression point by linearizing voltage and current at RF stages. The conversion gain of the mixer described in this work is 12 ± 1 dB at frequencies between 0.4 and 1.8 GHz, and the lowest SSB noise figure is 7 dB at 1 GHz. The measured noise figure is 7 dB ± 0.4 dB at frequencies between 0.4 and 1.8 GHz. The noise floor of the circuit is about −166 dBm, and IP3 and the 1 dB compression point are roughly 5 dBm and −8 dBm, respectively, so the available dynamic range is calculated as follows: (−174–1 dB compression point + NF). From this, (−174 − (−8) +7 ≈ 159 dBm/Hz) is the available dynamic range (however, Table 2 shows (−174 − (−13) + 18 ≈ 143 dBm).

In the intended band, the mixer achieves a good return loss of over 8 dB for an RF and LO port, and a measured return loss of over 18 dB at 145 MHz and IF frequency. Furthermore, the design achieved RF-to-IF isolation better than 35 dB and LO feedthrough and LO leakage isolation above 50 dB. Good linearity performance is demonstrated by the measured third-order intercept point of +4.7 dBm and the 1 dB compression point of roughly −8 dBm, respectively. Applications requiring low voltage can benefit from the suggested mixer architecture.

## Figures and Tables

**Table 1:** Calculated terms of the noise figure of the double-balanced mixer.

Terms	Calculated Values
$g_{m\_eff}=\frac{g_{m}}{1+g_{m}z_{E}}$	0.028628
$\frac{2}{{{g_{m}}^{2}R}_{L}}$	10.75013
${2*(r}_{b1}+\frac{1}{2g_{m}})$	68.67051
$\frac{2}{{g_{m}}^{2}}$	2440.28
$\left(2r_{b2}g_{m}+1\right)$	1.252272
$\frac{I_{RF}}{{\pi V}_{LO}}$	0.008954

**Table 2:** Comparison of several performance measures of the proposed double-balanced mixer.

References	[33]	[36]	[39]	[40]	In This Work
Device	130 nm	130 nm	180 nm	BJT	SiGe BJT
$f_{RF}$	2 GHz	900 MHz	3.432 GHz	935–960 MHz	0.5–2 GHz Test freq: 1 GHz
$f_{Lo}$	N/A	0.850 MHz	N/A	915 MHz	0.355–1.855 GHz Test freq: 855 MHz
$f_{IF}$	250 MHz	50 MHz	264 MHz	N/A	145 MHz
Degeneration Techniques	Feedforward linearization	Differential folded mixer with multiple feedback	Inductive degeneration	N/A	Series inductive and shunt-resistive feedback technique
Conversion Gain	8.5 dB	18.4 dB	11 dB	22.2 dB	12 dB ± 1 dB
Noise Figure (SSB)	18 dB	8.5 dB (DSB) 11.5 dB (SSB)	8 dB	N/A	7 dB (SSB)
1 dB Comp. Point	−13 dBm	−18 dBm		−25.5 dBm	−8 dBm
IIP3	~9 dBm (1 MHz spacing)	~11 dBm	0.257 dBm	−11.5 dBm	4.7 dBm (100 KHz spacing)

**Table 3:** Comparison between simulated and measured results after optimization.

Parameter Metrics	Simulated Performance	Measured Performance	Calculated Performance
RF Frequency	1 GHz	1 GHz	1 GHz
LO Frequency	855 MHz	855 MHz	855 MHz
Mixer Bandwidth	0.5 GHz to 2 GHz constant IF of 145 MHz	0.5 GHz to 1.8 GHz constant IF of 145 MHz	-
Conversion Gain Bandwidth	11.4 ± 0.5 dB	12 ± 1 dB	12.338 dB (predicted conversion gain)
Noise Figure	8 dB	7.03 dB	7.96 dB (predicted)
1 dB Compression Point	−6.9 dBm	Between −9 dBm and −8 dBm	-
IIP3 Measurement	6.716 dBm	4.7 dBm	-
OIP3 Measurement	17.9 dBm	15 dBm	
L–I Isolation	−65 dB	−58 dB	-
L–R Isolation	−65 dB	−70 dB	-
RF Port Return Loss	Better than −12 dB	Better than −8 dB	-
LO Port Return Loss	Better than −10 dB	Better than −8 dB	-
IF Port Return Loss	−26 dB at 145 MHz	−18 dB at 145 MHz	-

## Data Availability

Data are contained within the article.
